# Effect of national pre-ESRD care program on expenditures and mortality in incident dialysis patients: A population-based study

**DOI:** 10.1371/journal.pone.0198387

**Published:** 2018-06-01

**Authors:** Ming-Yen Lin, Li-Jen Cheng, Yi-Wen Chiu, Hui-Min Hsieh, Ping-Hsun Wu, Yi-Ting Lin, Shu-Li Wang, Feng-Xuan Jian, Chih Cheng Hsu, Shu-An Yang, Huei-Lan Lee, Shang-Jyh Hwang

**Affiliations:** 1 Division of Nephrology, Department of Internal Medicine, Kaohsiung Medical University Hospital, Kaohsiung Medical University, Kaohsiung, Taiwan; 2 Faculty of Renal Care, College of Medicine, Kaohsiung Medical University, Kaohsiung, Taiwan; 3 Department of Public Health, College of Health Science, Kaohsiung Medical University, Kaohsiung, Taiwan; 4 Graduate Institute of Clinical Medicine, College of Medicine, Kaohsiung Medical University, Kaohsiung, Taiwan; 5 Department of Family Medicine, Kaohsiung Medical University Hospital, Kaohsiung Medical University, Kaohsiung, Taiwan; 6 Department of Nursing, Kaohsiung Medical University Hospital, Kaohsiung Medical University, Kaohsiung, Taiwan; 7 Institute of Population Health Sciences, National Health Research Institutes, Miaoli, Taiwan; 8 Department of Health Services Administration, China Medical University, Taichung City, Taiwan; 9 Institute of Clinical Medicine, National Yang-Ming University, Taipei, Taiwan; Postgraduate Medical Institute, INDIA

## Abstract

Inadequate care of chronic kidney disease (CKD) is common and may be associated with adverse outcomes after dialysis. The nationwide pre-end-stage renal disease pay for performance program (P4P) has been implemented in Taiwan to improve quality of CKD care. However, the effectiveness of the P4P program in improving the outcomes of pre-dialysis care and dialysis is uncertain. We conducted a longitudinal cohort study. Patients who newly underwent long-term dialysis (≥3 mo) between 2007 and 2009 were identified from the Taiwan National Health Insurance Research Database. Based on the patient enrolment of the P4P program, they were categorized into P4P or non-P4P groups. We analysed pre-dialysis care, healthcare expenditures, and mortality between two groups. Among the 26 588 patients, 25.5% participated in the P4P program. The P4P group received significantly better quality of care, including a higher frequency of glomerular filtration rate measurement and CKD complications survey, a higher rate of vascular access preparation, and more frequent use of arteriovenous fistulas than the non-P4P group did. The P4P group had a 68.4% reduction of the 4-year total healthcare expenditure (excluding dialysis fee), which is equivalent to US$345.7 million, and a significant 22% reduction in three-year mortality after dialysis (hazard ratio 0.78, 95% confidence interval: 0.75–0.82, *P* < 0.001) compared with the non-P4P group. P4P program improves quality of pre-dialysis CKD care, and provide survival benefit and a long-term cost saving for dialysis patients.

## Introduction

Chronic kidney disease (CKD) is a crucial public health concern worldwide. The number of patients receiving renal replacement therapy is projected to steadily increase from 2.618 million in 2010 to 5.439 million in 2030 worldwide, mostly in developed countries[1]. With growing patient population and financial burden, several treatment and financing strategies have been developed to ensure that appropriate care is provided and morbidities, mortality and expense are under control. Pay-for-performance (P4P) is a promising approach to improving the quality of health care by linking financial incentives to provider’s performance. The rationale behind the initiative is through explicitly paying for recommended care, improvements in quality can be promoted, leading to better patient outcomes. For example, the Centers for Medicare & Medicaid Services in the United States launched a mandatory federal P4P program in 2012 to encourage provision of better anemia management, dialysis adequacy, and use of arteriovenous fistula to patients with end-stage renal disease (ESRD) [2]. Similar P4P programs for complication monitoring, risk factor control, and preparation for renal replacement in pre-dialysis phase are also adopted in other countries [3–7]. In Taiwan, a nationwide pre-ESRD P4P care programme was launched to provide more comprehensive care to patients with advanced CKD in 2006. Under this incentive scheme, nephrologists are requested to provide care not only more closely following clinical guidelines, also based on a multidisciplinary team [4].

Multidisciplinary care (MDC) consists of various healthcare professionals, which has been widely adopted in the provision of CKD treatment. Previous studies demonstrated that MDC in CKD was associated with more effective medication, a lower renal progression rate, and a decreased risk of temporal catheterization for dialysis [8–12]. Although studies also suggested that MDC may help contain costs of CKD care [4, 11, 13, 14] and improve quality in early phase of dialysis [15], whether a MDC under P4P scheme continuously improves dialysis outcomes and resource use remains to be examined.

This study aimed to investigate whether the nationwide pre-ESRD P4P program in Taiwan improves the quality of CKD care, risks of all-cause and specific mortality, and long-term resource use and healthcare expenditure after dialysis. We conducted a cohort study at population level to examine the long-term effect of the pre-ESRD P4P program after dialysis on enrolled P4P patients, compared with patients not participating in the P4P program.

## Materials and methods

### Overview of Taiwan pre-ESRD P4P program

Taiwan National Health Insurance (NHI) is a mandatory, universal, single-payer insurance system introduced in 1995 and covers over 99% of the residents in Taiwan. In 2006, The NHI launched a P4P scheme with indicators for pre-ESRD care to encourage nephrologists to cooperate with nurse and dietitian to provide MDC for patients with CKD stage 3–5. The important features of this program has been described in previous work [4]. In brief, patients can voluntarily enrol in this program and after enrolment, will be requested to receive comprehensive assessments and patient education. The MDC team will receive a bonus if they achieve pre-specified quality indicators. The indicators include CKD management, patient education, continuous care, remission of proteinuria, and maintenance of renal function (S1 File).

### Design and setting

We conducted a retrospective cohort study using the P4P Registry linked with NHI claim database. The database is anonymous and contains detailed information on outpatient, inpatient, and emergency claims [16]. All incident ESRD patients who initiated dialysis between 2007 and 2009 and had been on dialysis for more than 3 months were identified based on the International Classification of Diseases, Ninth Revision, Clinical Modification (ICD-9-CM) codes in the Registry of Catastrophic Illness (Table A in S1 File). Patients were assigned to the P4P group if they had enrolled in the P4P program before dialysis; they were assigned to the Non-P4P group otherwise. Dates of the first dialysis were retrieved from first appearance of each patient’s specific payment code (Table B in S1 File). The information of identified cohort was collected in a year before dialysis therapy and after 3 years of follow-up. The ethical review board of Kaohsiung Medical University Hospital approved this study (KMUHIRB-EXEMPT (I)-20150062). All research procedures followed the directives of the Declaration of Helsinki.

### Study outcomes

The study outcomes were the quality of pre-dialysis care, health care utilisation, health care expenditure, and mortality. Indexes for quality of pre-dialysis care were defined as the frequency of estimated glomerular filtration rate (eGFR) measurement, screening for CKD-related complications (e.g., potassium, calcium and phosphorus), vascular access preparation before dialysis, the proportion of outpatient dialysis initiation, dialysis initiation without hospitalisation, use of a temporary catheter, and use of an arteriovenous fistula (AVF) as the primary vascular access, and obtained by tracing the pre-specified payment codes (Tables A and B in S1 File). Health care utilisation was assessed according to the annual number of outpatient visits, emergency visits, inpatient visits and the length of stay. Health care expenditure included the annual expenses of outpatient visits (excluding expenses for dialysis), inpatient visits and all medical visits (excluding expenses for dialysis) incurred a year before and 3 years after the initiation of dialysis. For precise reflection measurement of expense difference between groups, an equal discount rate 3% was applied in the cost analysis. All expenses were reported as US dollars (in 2012, US $1 = New Taiwan $ 30). NHI reimbursement is based on the number of points conferred to each care under the global budget floating value system. On average, a point equals to 0.9 New Taiwan Dollar (NTD) between 2007 and 2012. Death was ascertained by reason of withdrawal from NHI claim database. Death within the 3-year follow-up period was considered an event of interest; those who received renal transplantation or survived at the end of the follow-up period were considered censored. Furthermore, the causes of mortality after dialysis, such as cardiovascular diseases, infectious diseases, and cancer, were assessed according to the primary diagnosis codes entered in inpatient claims and emergency claims 30 days before death (Table A in S1 File).

### Covariates of interest

Covariates used in the model were patient demographics (age and sex), socioeconomic status reported in the Registry for Beneficiaries (dependent, earning < NTD 20 000, and earning ≥ NTD 20 000 in 2006), urbanisation level of residence, modality (haemodialysis or peritoneal dialysis), comorbidities (diabetes mellitus, hypertension, cardiac disorders, ischemic stroke, gout, and peripheral vascular disease), and Charlson comorbidity index (CCI). Occurrence of comorbidity was defined according to the appearance of the corresponding ICD-9-CM diagnosis code or procedure code (Table A in S1 File) more than twice in outpatient claims or more than once in inpatient claims a year before dialysis initiation. CCI was calculated according to the diseases listed in a previous study [17]. Furthermore, antidiabetic drugs, antihypertensive drugs, analgesic drugs, antilipidaemic drugs, and erythropoietin prescribed a year before dialysis were included in the model to manage confounding effect (Table C in S1 File).

### Statistical analysis

Distributions of characteristics were presented as mean ± standard deviation, median (interquartile range), or percentage. Differences between groups were analysed using independent *t* test, Wilcoxon sum-rank test, and chi-square test. Annual health care utilisation and expenditure were presented as the mean ± standard deviation. The effects of the P4P program on repeated measurements of outcome measures over time was evaluated using generalized linear mixed model. Because fitting complicated model to our massive data can be difficult, we simplified the model by assuming normal distribution of the random effects. Previous studies had indicated that little bias in estimation of covariates effects can be obtained even misspecification of the distribution occur [18, 19]. Coefficients were estimated using robust standard errors to correct misspecification of the correlation structures. Data were represented as adjusted difference and their 95% confidence intervals (CI) between P4P and Non-P4P groups. 3-year cumulative survival rate after dialysis was estimated using the Kaplan–Meier approach. Differences between two groups were assessed using the log-rank test. We also used propensity score (PS) matching approach to add comparability between two groups. The PS was calculated by considering all covariates as independent variables through multiple logistic regression. Multivariable analysis with adjustment of all covariates was conducted for all patients and univariable analysis for PS matched patients only, using Cox regression hazard models to obtain hazard ratio (HR) and 95% CI. A series of sub-analyses stratified by age, sex, modality, and comorbidities was conducted to determine the association between P4P enrolment and mortality risk. Because death from other cause may play a role on the estimation of cause-specific mortality, subdistribution hazard model was used to determine the effects of the P4P program on specific mortality [20]. Statistical analyses were performed using SAS 9.3 software (SAS Institute Inc., Cary, NC, USA), and figures were created using GraphPad Prism 5.0 (GraphPad Software Inc., San Diego, CA, USA). A 2-sided *P* value <0.05 indicated statistical significance.

## Results

### Demographic characteristics

We identified 27 293 patients who initiated long-term dialysis between 2007 and 2009. Patients aged <18 years (n = 152), those who received post transplantation renal care as registered in the Resisted Catastrophic Illness (n = 550), and those who lacked blood access records in their claims data (n = 3) were excluded (Fig A in S1 File). Consequently, 26 588 patients were eligible for follow-up. Patients enrolled in the P4P program accounted for 25.5% of the entire study cohort. Median duration of patient enrolled this program was 309 days (interquartile range, 170–503 d). The P4P group was more likely to be in a higher socioeconomic status, select peritoneal dialysis, have more major coexisting diseases, and use more medications (Table 1). After PS matching, these characteristics in the 2 groups were balanced except for the percentage of antihypertensive drugs use.

**Table 1:** Characteristics of the study cohort.

	Overall n = 26 588	Before PS matching		P value	After PS matching		P value
		Non-P4P n = 19 807	P4P n = 6781		Non-P4P n = 6776	P4P n = 6776	
Age, mean±SD, y	63.2±14.0	63.1±14.3	63.3±13.2	0.22	63.5±13.5	63.3±13.2	0.33
Female sex (%)	48.4	48.0	49.6	0.03	49.9	49.6	0.71
Socioeconomic status, %, NT dollar				<0.001			0.95
Dependent	34.2	34.3	34.1		34.1	34.1	
<20,000	21.8	22.6	19.3		19.2	19.4	
≧20,000	44.0	43.1	46.6		46.7	46.5	
Urbanization, %				0.82			0.84
Rural	29.4	29.4	29.3		29.4	29.3	
Urban	70.6	70.6	70.7		70.6	70.7	
Modality, %				<0.001			0.72
Hemodialysis	85.7	87.1	81.6		81.9	81.6	
Peritoneal dialysis	14.3	12.9	18.4		18.1	18.4	
Major coexisting disease, %							
Diabetes	59.0	58.6	59.9	0.07	60.7	59.9	0.30
Hypertension	89.2	88.4	91.5	<0.001	92.5	91.5	0.03
Cardiac disorder	25.9	26.6	23.9	<0.001	24.1	23.9	0.73
Ischemic stroke	8.8	9.1	7.8	<0.001	7.9	7.8	0.82
Gout	17.2	16.7	18.7	<0.001	18.5	18.7	0.84
Peripheral vascular diseases	5.4	5.5	5.1	0.21	5.0	5.1	0.94
Confounding drugs, %							
Diabetic drugs	51.0	50.4	53.0	<0.001	54.0	53.0	0.21
Antihypertensive drugs	89.8	88.1	94.8	<0.001	96.2	94.8	0.0002
NSAID	13.8	14.5	11.7	<0.001	12.1	11.7	0.47
Anti-lipid drugs	27.3	25.8	31.6	<0.001	31.6	31.6	0.97
Erythropoietins	46.8	40.0	67.0	<0.001	66.1	66.9	0.31
Charlson comorbidity index							
Mean±SD	4.7±2.1	4.7±2.1	4.7±2.1	0.92	4.7±2.0	4.7±2.1	0.35
Median (IQR)	3 (5–6)	3 (5–6)	3 (5–6)	0.88	3 (5–6)	3 (5–6)	0.31

Abbreviation: PS, propensity score; P4P, pay for performance; NSAID, nonsteroidal anti-inflammatory drugs.

Chi-square test, independent t test, and Wilcoxon rank-sum test were used to test the differences between the P4P group and the Non-P4P group for categorical and continuous variables. Statistical significance is defined as p value less than 0.05.

### Quality of pre-dialysis care

**Fig 1:**
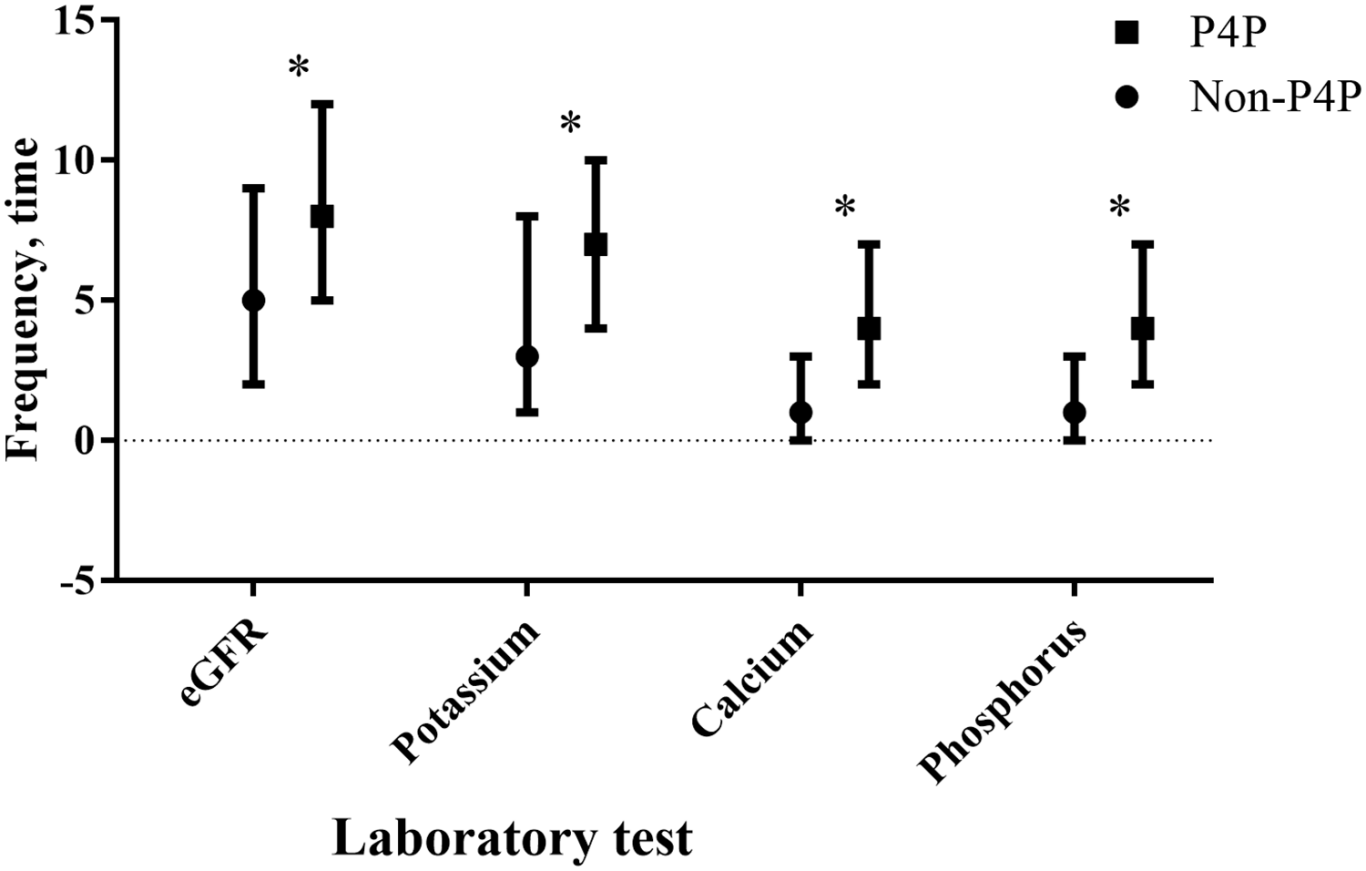
Quality of predialysis care by the pre-end-stage renal disease. Pay-for-performance (P4P) Program Enrolment. Frequency of estimated glomerular filtration rate (eGFR) monitoring and chronic kidney disease complications survey one year before dialysis were compared between the P4P and Non-P4P groups, and *P <0.001 were showed according to the Wilcoxon rank-sum test. “┬” and “┴” represent as 75 and 25 percentiles, respectively.

**Fig 2:**
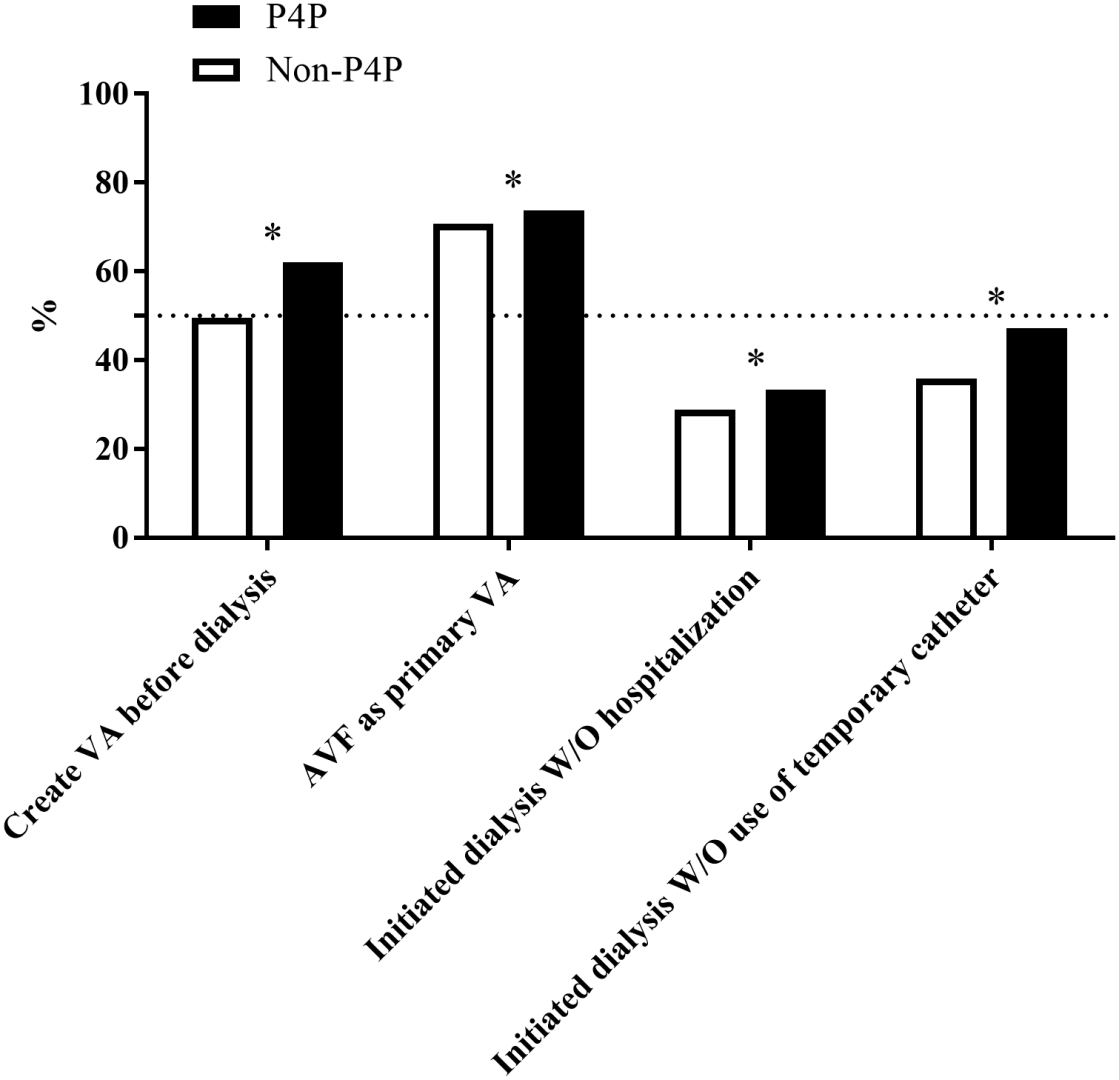
Dialysis preparation by the pre-end-stage renal disease pay-for-performance (P4P) program enrolment. Data are represented as the percentage, and the differences between the two groups were analysed using the chi-square test. Abbreviation: VA, vascular access; W/O, without.

Patient in the P4P group received more frequent measurements of renal function (median [interquartile range] = 8 [5–12] vs 5 [2–9], *P* < 0.001) and more laboratory tests for screening CKD-related complications in one year before dialysis. The P4P group had more frequent potassium (7 [4–10] vs 3 [1–8], *P* < 0.001), calcium (4 [2–7] vs 1 [0–3], *P* < 0.001), and phosphorus (4 [2–7] vs 1 [0–3], *P* < 0.001) measurements than those in Non-P4P group (Fig 1). In addition, the P4P group was better prepared with vascular access creation before dialysis initiation (62.0% vs 49.5%, *P* < 0.001), selected an AVF as the primary vascular access for haemodialysis more frequently (73.8% vs 70.7%, *P* < 0.001), initiated dialysis without hospitalisation (33.4% vs 28.8%, *P* < .001), and used a temporary catheter less frequently (52.8% vs 64.2%, *P* < 0.001) (Fig 2).

### Health care utilization and expenditure

Table 2 illustrates the differences in mean change of annual health care utilisation and expenditure between two groups in mixed-effects model analysis. After adjusted all covariates, patient who enrolled in P4P program were significantly associated with a 1.8 times increase in annual outpatient visits, a 105 USD increase in annual outpatient expenses, and a slightly 0.09 times increase in annual emergency. However, a significantly shorter length of stay (−1.7 d in average, *P* < 0.001), and lower annual inpatient expenses (−404 USD in average, *P* < 0.001) were also observed in the P4P group, compared with the Non-P4P group, contributing to a lower per patient total annual health care expenses by 318 USD (95% CI = 151–484, *P* < 0.001) in the P4P group. In other word, the P4P group had a 68.4% reduction of the 4-year total healthcare expenditure (excluding dialysis fee), which is equivalent to US$345,691,751.

**Table 2:** Effects of the pre-end-stage renal disease pay-for-performance program on resource utilization and health care expenditure.

Measures Group	Pre-dialysis	Post dialysis			Adjusted difference in increase (95% CI)	P value
	Year 1	Year 1	Year 2	Year 3		
Outpatient visit, time						<0.001
Non-P4P	28.7±17.8	24.5±16.2	23.6±17.8	23.8±18.3	0 [Reference]	
P4P	32.9±17.2	26.3±16.2	25.6±18.0	26.1±18.8	1.8 (1.4; 2.1)	
Outpatient expenses, (excluding dialysis fee)						<0.001
Non-P4P	1985±1894	1615±2123	1387±1665	1320±1579	0 [Reference]	
P4P	2637±1443	1647±1532	1453±1672	1419±1743	105 (64; 145)	
Emergency visit, time						0.002
Non-P4P	1.8±2.6	2.1±2.8	1.5±2.7	1.6±3.2	0 [Reference]	
P4P	1.8±2.4	2.0±3.1	1.6±3.2	1.7±3.7	0.09 (0.04; 0.15)	
Hospital admission, time						0.58
Non-P4P	2.2±1.8	2.3±2.1	1.1±1.8	1.1±1.8	0 [Reference]	
P4P	2.1±1.7	2.1±1.9	1.1±1.7	1.1±1.7	-0.01 (-0.04; 0.02)	
Length of stay, day						<0.001
Non-P4P	28.3±32.4	29.2±44.0	12.9±34.9	12.3±33.7	0 [Reference]	
P4P	22.1±24.3	23.4±32.4	11.6±32.3	11.2±28.7	-1.7 (-2.3; -1.0)	
Inpatient expenses						<0.001
Non-P4P	6002±8084	6913±10170	3278±8557	3052±8184	0 [Reference]	
P4P	4455±5448	5580±8175	3036±7884	2906±7202	-404 (-557; -251)	
Overall health care expenses (excluding dialysis fee)						<0.001
Non-P4P	8275±8385	9027±10913	5025±8964	4736±8610	0 [Reference]	
P4P	7371±5649	7680±8621	4840±8344	4697±7773	-318 (-484; -151)	

Abbreviation: P4P, pay for performance; CI, confidence interval.

Data displays as mean ± standard deviation.

All expenses were reported as US dollars (in 2012, US $1 = New Taiwan $ 30). NHI paid to health care provider using point under the global budget floating value system, in which a point equals to 0.9 New Taiwan Dollars between 2007 and 2012. For precise reflection measurement of expense difference between groups, an equal discount rate 3% was applied in the cost analysis.

Parameter was estimated using generalized mixed model with link function of normal distribution after adjusted time, age, sex, socioeconomic status, urbanization, modality, major coexisting disease (diabetes, hypertension, cardiac disorder, ischemic stroke, gout, peripheral vascular diseases), Charlson score, and confounding drugs (diabetic drugs, anti-hypertensive drugs, analgesic drugs, anti-lipid drugs, and erythropoietins).

### Three-year cumulative survival and hazard ratios of mortality

**Fig 3:**
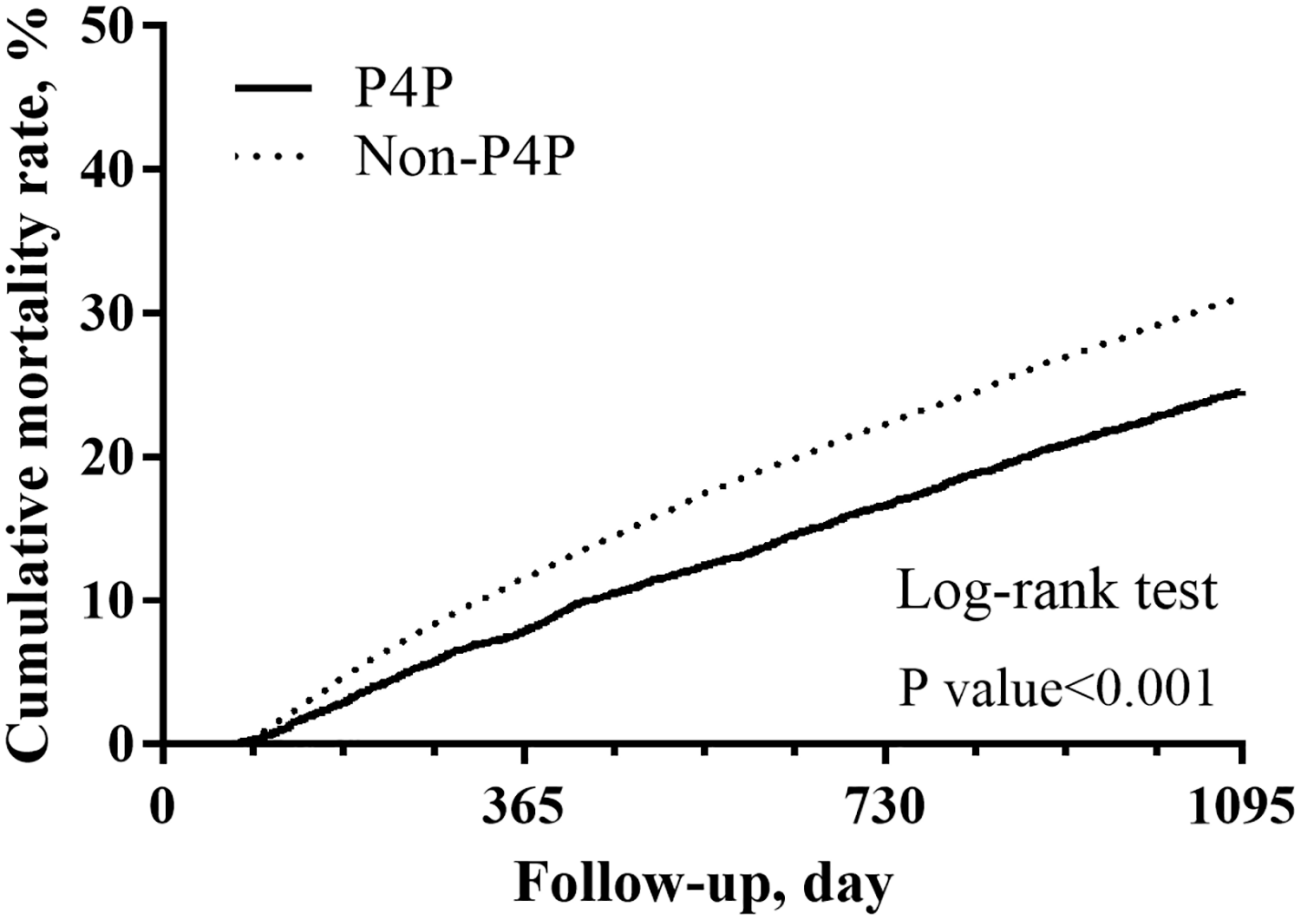
Cumulative three-year survival rate. Data were estimated using the Kaplan–Meier approach, and the differences in the survival rate between the pre-end-stage renal disease pay-for-performance (P4P) and Non-P4P groups were compared using the log-rank test.

**Fig 4:**
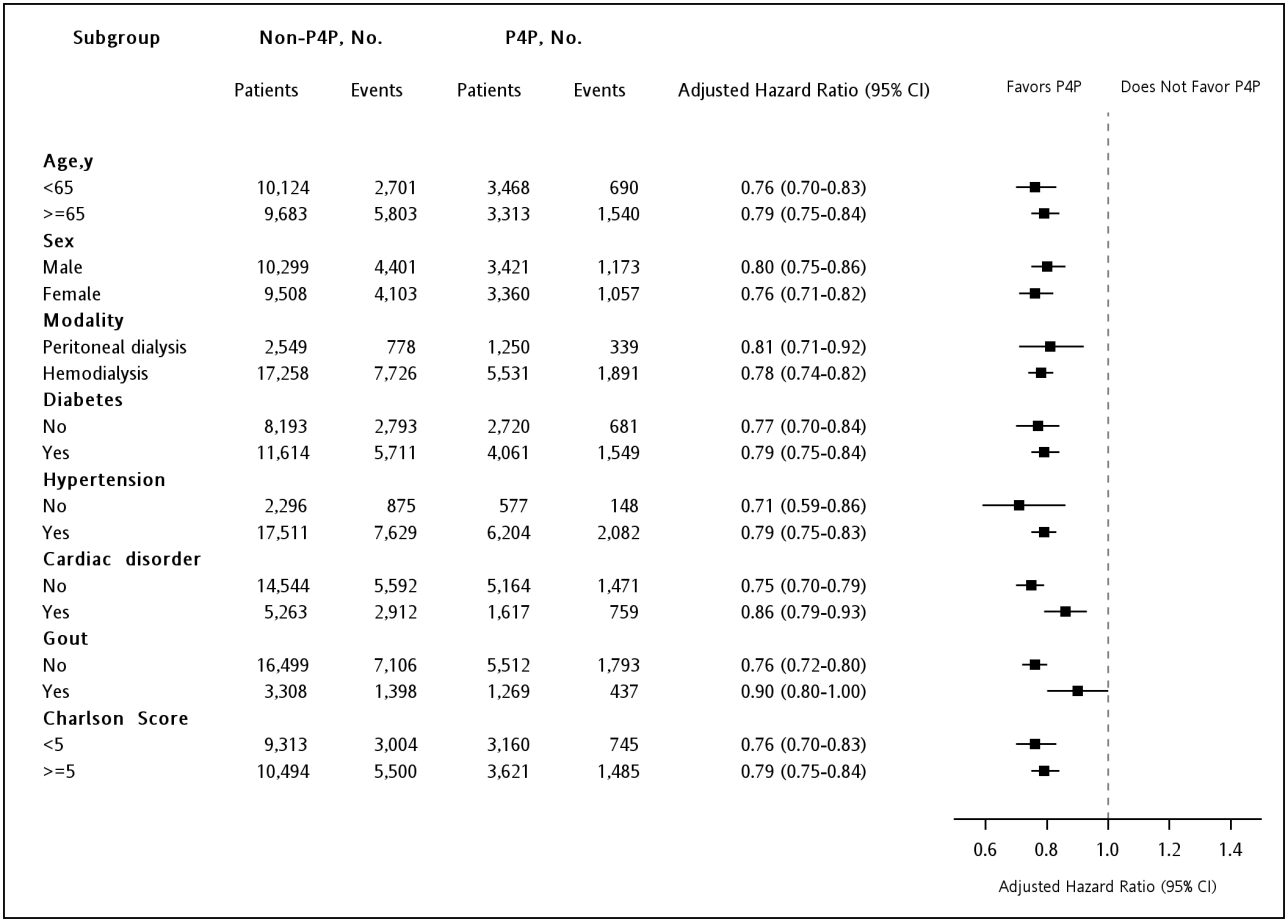
Multivariable stratified analyses of the association between the pre-end-stage renal disease pay-for-performance (P4P) program and mortality. The hazard ratio (HR) and 95% confidence interval (CI) of the difference in mortality risk between the P4P and Non-P4P groups were determined using multivariable Cox regression hazard models based on adjusted all other covariates.

Fig 3 depicts the differences in the cumulative survival rate between two groups. During the study period, mortality after dialysis was significantly decreased in the P4P group compared with the Non-P4P group (cumulative survival rate = 75.3% vs 68.8%, *P* < 0.001). Regression results showed that enrolment in the P4P program was associated with a lower three-year mortality risk after dialysis (all eligible patients, adjusted HR = 0.78, 95% CI = 0.75–0.82; PS-matched patients, HR = 0.77, 95% CI = 0.73–0.82). A similar trend was observed for mortality due to out-of-hospital cardiac arrest (adjusted HR = 0.68, 95% CI = 0.57–0.80). Mortality attributed to cardiovascular diseases (PS-matched HR = 0.79, 95% CI = 0.64–0.96) and infectious diseases (adjusted HR = 0.85, 95% CI = 0.77–0.93), but not cancer (adjusted HR = 0.90, 95% CI = 0.72–1.13), was also lower in the P4P group than in the Non-P4P group (Table 3). Finally, the results of all stratified analyses revealed consistently lower mortality in the patients enrolled in the P4P program than in those who were not (Fig 4).

**Table 3:** Effects of the pre-end-stage renal disease pay-for-performance program on the 3-year mortality after dialysis.

	Events (Incidence per 1,000 patient year)	Adjusted hazard ratio (95%CI)			PS-matched hazard ratio (95%CI)
		Model 1	Model 2	Model 3	Model 4
All cause					
Non-P4P	8485 (171.6)	1.00 [Reference]	1.00 [Reference]	1.00 [Reference]	1.00 [Reference]
P4P	2216 (125.1)	0.71 (0.67–0.74)	0.71 (0.68–0.74)	0.78 (0.75–0.82)	0.77 (0.73–0.82)
Cause of death					
Cardiovascular disease					
Non-P4P	598 (12.1)	1.00 [Reference]	1.00 [Reference]	1.00 [Reference]	1.00 [Reference]
P4P	117 (10.0)	0.86 (0.73–1.02)	0.85 (0.72–1.00)	0.86 (0.73–1.03)	0.79 (0.64–0.96)
Infectious disease					
Non-P4P	2331 (47.1)	1.00 [Reference]	1.00 [Reference]	1.00 [Reference]	1.00 [Reference]
P4P	609 (34.4)	0.74 (0.68–0.81)	0.75 (0.69–0.82)	0.85 (0.77–0.93)	0.81 (0.72–0.90)
Cancer					
Non-P4P	372 (7.5)	1.00 [Reference]	1.00 [Reference]	1.00 [Reference]	1.00 [Reference]
P4P	110 (6.2)	0.88 (0.71–1.09)	0.88 (0.71–1.10)	0.90 (0.72–1.13)	0.86 (0.67–1.11)
Out-off-hospital cardiac arrest					
Non-P4P	770 (15.6)	1.00 [Reference]	1.00 [Reference]	1.00 [Reference]	1.00 [Reference]
P4P	178 (10.0)	0.66 (0.56–0.78)	0.66 (0.56–0.78)	0.68 (0.57–0.80)	0.65 (0.54–0.79)

Abbreviation: P4P, pay for performance; CI, confidence interval; PS, propensity score.

Subdistribution hazard model was used to estimate cause-specific mortality after consideration of competing risk of other causes.

Model 1: adjusted patient characteristic (age, sex, socioeconomic status, urbanization, and modality).

Model 2: adjusted factors listed in model 1+major coexisting disease (hypertension, diabetes, cardiac disorder, ischemic stroke, gout, and peripheral vascular diseases), and Charlson score.

Model 3: adjusted factors listed in model 2+ confounding drugs (diabetic drugs, anti-hypertensive drugs, analgesic drugs, anti-lipid drugs, erythropoietins).

Model 4: analysis only in patients with propensity score matching.

## Discussion

Despite the importance of advanced-stage CKD care to clinical and economic outcomes has been well recognised, improvement of care quality remains a great challenge for policymakers. To our knowledge, this paper is the first study to evaluate the effect of a national pre-ESRD P4P program on patients who underwent dialysis. The results suggested that the P4P program implemented with a multidisciplinary team care model has significantly improved the quality of CKD care, reduced the early mortality risk, and lowered heath care expenditures.

Although the frequency of eGFR monitoring and the evaluation of CKD-related complications have been suggested by guidelines in past decades [21, 22], the adherence of these guidelines in clinical practice has not been comprehensively investigated. Without regular evaluation of eGFR and CKD complications, it may be difficult for nephrologists to provide patients with appropriate treatment. Even under pre-ESRD nephrology care, the quality of care is not completely acceptable. A recent study found that only 37% of dialysis institutes that intensively performed pre-ESRD nephrology care optimally responded to anaemia and prepared an AVF for dialysis [23]. Our findings suggested that a well-designed care model, supported by appropriate financial incentives, may help physicians recognise the importance of detection and outcome monitoring and thus encourage them to adopt a more active CKD care.

The main target of pre-ESRD care is reduction of overall morbidity and mortality by appropriate prevention of complications, timely preparation for renal replacement therapy, and conservative care. A key element is early referral to a nephrologist, which has been considered beneficial for prognosis. For example, a systematic review that investigated the results of 22 observational studies that explored the effect of timing of nephrology referral on CKD outcomes, and highlighted an association of late referrals with increased morbidity and mortality [24]. On the other hand, early referral does not necessarily lead to a good quality of care. A previous study reported that number and consistency of care by a nephrologist in 3 months or more of the 6 months before dialysis were directly associated with a superior prognosis [25]. A decreasing number of nephrology visits is associated with a poor quality of care and adverse outcomes. Under P4P programs, the process of care and intermediate outcomes associated with clinical quality are rewarded, offering incentives to physicians to create a mature AVF at the most appropriate time. This explains why the P4P group in this study was significantly less likely to initiate dialysis with a temporary catheter and to be hospitalised than the Non-P4P group.

The economic evaluation of various healthcare programs in CKD care has not been widely applied. Previous studies evaluating early referrals to nephrologists and MDC were mostly based on small samples and a short follow-up time and have reported a significant cost-saving effect [11, 13–15]. Our findings indicated that the reduction in healthcare expenses remained substantial 3 years after dialysis. In P4P programs, it may be helpful for both physicians and patients with CKD to monitor and manage the disease progression closely if more outpatient visits are scheduled, thereby reducing the risk of emergency and inpatient visits and total health care costs. Further investigation of the cost-effectiveness of similar incentive programs of pre-ESRD care may facilitate informing policymakers about the link between direct financial incentives and improvement of the quality of care.

The average mortality risk was 22% lower in the P4P group than in the Non-P4P group. Monitoring of CKD-related complications and dialysis preparation may explain this lowered mortality risk. For instance, P4P patients under regular potassium and phosphate measurement were more likely to receive appropriate medications before vascular calcification occurred, thus reducing the risk of cardiovascular diseases. Moreover, care providers tended to prepare an AVF creation before dialysis initiation for patients in the P4P group. Current guidelines for dialysis highly recommend an AVF for its lasting patency and low risk of infection [26, 27]. Poor health outcomes were significantly associated with the use of a catheter and graft, which were more frequently observed in the non-P4P patients. Artificial material of catheter and graft may cause chronic inflammation and bacteraemic infection [28], increasing the mortality risk [29]. Their shorter patency is often associated with a greater probability of invasive procedures, such as thrombectomy and percutaneous transluminal angioplasty, and exposes patients to an excessive mortality risk.

This research has several policy implications. First, this is one of few study to investigate the effectiveness of a national P4P program of multidisciplinary pre-ESRD care. Previous studies have either analysed other chronic diseases or evaluated MDC without explicit financial incentives. Second, the quality of pre-ESRD care can be greatly improved by aligning MDC with financial incentives. Third, total health care expenditures can be significantly lowered by emphasising preventive services such as eGFR measurement, complication screening, and well-planned dialysis. The mortality risk after dialysis can be affected by the care delivered at the pre-ESRD stage.

This study has several limitations. First, although the PS matching method balanced the distributed difference of variables available in the dataset, there could be confounding effects generated by unidentified variables. Second, lack of laboratory data made identification of patients who received dialysis due to acute kidney injury impossible. Such patients, although small in number, were not the target population of this study. Third, we could not obtain the real cause of death. The causes were identified using diagnosis codes reported in inpatient claims a month before the mortality date. The causes of death in patients who did not die during hospitalisation could not be determined, resulting in possible underestimation of mortality or misclassification of the causes of death. However, previous studies reported that mortality causes identified by using similar method were highly accurate [30, 31]. The impact to our results was considered minimal. Finally, the data were obtained from dialysis population in Taiwan. The results may not be generalizable to other countries.

In conclusion, our results demonstrated that under a MDC model supported by P4P scheme, patients received a higher-quality nephrology care, incurred lower healthcare costs, and exhibited longer overall survival. These findings are informative for policymakers and physicians to consider whether a physician incentive system helps improve the quality and outcomes of the current health care delivery model for patients with CKD.

## Supporting information

S1 File Pre-end-stage renal disease pay-for-performance program in Taiwan. (DOCX)
