# Supplementary material for: Effect of national pre-ESRD care program on expenditures and mortality in incident dialysis patients: A population-based study
Source: PLoS One. 2018 Jun 1;13(6):e0198387. doi: 10.1371/journal.pone.0198387 (PMC5983494; doi:10.1371/journal.pone.0198387)

**Supplementary Material**

**Key Points**

**Introduction of Pre-End-Stage Renal Disease Pay-for-Performance Program in Taiwan**

**Table A**. **The corresponding ICD-9-CM codes for the diagnosis of disease in the study.**

**Table B. The payment codes for the clinical treatment providing by Taiwan National Health Insurance.**

**Table C. Anatomical Therapeutic Chemical codes of drugs used concomitantly by patients during the study period.**

**Fig. A Study flow diagram.**

**Pre-End-Stage Renal Disease Pay-for-Performance Program in Taiwan**

National Health Insurance (NHI) is a mandatory, universal, single-payer insurance system introduced in 1995 and covers more than 99% of the residents in Taiwan. Since 2006, the pay-for-performance (P4P) program has become a component of reimbursement in care delivery to patients with chronic kidney disease (CKD) at stage 3b or higher under NHI. The pre-end-stage renal disease (Pre-ESRD) P4P program was designed through patient-centred case management by a multidisciplinary team for improving the quality of care for high-risk CKD patients to prevent or delay dialysis, avoid uremic complications, and restrain increasing health care costs. All contracted providers with a multidisciplinary CKD care team consisting of nephrologists, nurses, and dieticians were invited to participate. CKD patients who are at stages 3b, 4, and 5 or those with proteinuria (urine protein to creatinine ratio [UPCR] > 1000 mg/g) are eligible for enrolment. Case management services are terminated for enrolled patients who initiate long-term dialysis, receive kidney transplantation, achieve complete remission of proteinuria (UPCR < 200 mg/g), receive hospice care, or lose contact (eg, loss to followup, transfer to other health facilities, or death).

In P4P, health care providers receive additional bonus payments for patient enrolment, comprehensive patient education, annual evaluation, and 4 types of case management. Moreover, they are encouraged to achieve the minimal level of a detailed set of quality indicator targets, depending upon the health status of an enrolled patient with CKD. The quality indicator targets include prespecified process (eg, blood pressure < 130/80 mmHg, total cholesterol/triglyceride < 200 mg/dL, serum albumin > 3.5 g/dL, HbA1c < 7.5%, and haematocrit > 28%) and intermediate outcome measures (eg, creation of vascular access before dialysis and use of erythropoietin, peritoneal dialysis, and outpatient dialysis). To claim the bonus payments, the providers must collect and report data on the quality indicators to the NHI within 3 months before or after physician visits. Quality bonuses are disbursed according to the provision of multidisciplinary team care and attained process and outcome dimensions of care. Each quality indicator is rewarded with prespecified points that are converted to payments for providers on a quarterly basis. The monetary value of each point is <1 New Taiwan dollar; however, its actual value is determined as per the available budget. The reimbursement policy is described in detail as follows.

1. Bonus Payment for Initial Enrolment Visits (P3402C)

Participating providers are awarded 1200 points for offering the following services to each enrolled eligible patient with CKD: physician care (400 points), nursing care (200 points), dietician service (200 points), and data management (400 points).

1. Bonus Payment for Comprehensive Patient Education (P3403C)

Participating providers are rewarded 600 points for providing comprehensive education and dietician services at each follow-up visit. Claims for payment can be submitted every 3 months since the 77th day after initial enrolment.

1. Bonus Payment for Annual Evaluation Visits (P3404C)

Participating providers are rewarded 600 points for annual physical examinations after 4 comprehensive follow-up education visits (P3403C) are completed within 2 years. This payment can be claimed once a year for each enrolled patient.

1. Bonus Payment for Closing the Case (P3405C)

Participating providers are rewarded 600 points for closing a case under the following conditions: (a) following CKD patients at stage 5 for more than 3 months (at least one claim is mandatory) and (b) following CKD patients at stage 3b or 4 or those with proteinuria for more than 6 months (at least 2 claims are mandatory).

1. Bonus Payment for Case Management (P3406C)–CKD patients at stages 3b and 4

In total, 1500 points are rewarded for caring for enrolled CKD patients at stage 3b or 4 if their estimated glomerular filtration rate (eGFR) values are reduced to less than 4 mL/min/1.73 m^2^ after a year under the P4P program. This payment is made for each qualified case annually and is reviewed every year during the eligible period.

1. Bonus Payment for Case Management (P3407C)–CKD patients at stage 5

In total, 3000 points are rewarded for caring for enrolled CKD patients at stage 5 who achieve either of the following 2 conditions after a year under the P4P program: (a) patients do not initiate dialysis and reductions in their eGFR are less than 6 mL/min/1.73 m^2^, and (b) patients initiate long-term dialysis or receive kidney transplantation, but reductions in eGFR values are less than 6 mL/min/1.73 m^2^ and vascular access is created before dialysis. This bonus payment is made for each qualified case annually and is reviewed every year during the eligible period.

1. Bonus Payment for Case Management (P3408C)–CKD patients with proteinuria

Participating providers are rewarded 1000 points for caring for enrolled CKD patients with proteinuria, if they achieve complete remission of proteinuria (UPCR < 200 mg/g) after a year under the P4P program. This bonus payment is made for each qualified case and can be claimed only once.

1. Bonus Payment for Case Management (P3409C –Continual care

In total, 2000 bonus points are rewarded for caring for CKD patients at stage 3b, 4, or 5 and those with proteinuria, if they participate in the P4P program for 3 years and their claims for bonus payment for comprehensive patient education have been submitted 4 times within 2 years. The payment can be claimed for each eligible patient once a year after being qualified.

**Table A. The corresponding ICD-9-CM codes for the diagnosis of disease in the study**

| **Disease** | **Database^#^** | | **Corresponding ICD-9-CM codes** |
| --- | --- | --- | --- |
| Diabetes | CD/DD | | 250 |
| Hypertension | CD/DD | | 401-405 |
| Cardiac disorder | CD/DD | | 410, 428 |
| Ischemia stroke | CD/DD | | 433, 434, 436 |
| Gout | CD/DD | | 274 |
| Peripheral vascular diseases | CD/DD | | 440.2- 440.4, 443.9 |
| **Identified patients form the Registry for Catastrophic Illness Patient Database** | | | |
| Chronic renal failure under regular dialysis: | | | |
| End-stage renal disease | | HV | 585 |
| Hypertensive heart or renal disease with renal failure | | HV | 403.01, 403.11,  403.91, 404.02,  404.03, 404.12,  404.13, 404.92, 404.93 |
| Renal transplantation | | HV | V42.0 |
| **Identified patient’s cause of death form inpatient and emergency visit claim data** | | | |
| Cancer | CD/DD | | 140-208 |
| Cardiovascular disease | CD/DD | | 410-411, 433-434, 436, 430-432 |
| Infectious disease: | CD/DD | |  |
| Sepsis | CD/DD | | 038 |
| Infective endocarditis | CD/DD | | 421.0-421.1, 421.9 |
| Pneumonia | CD/DD | | 480-487, 507, 518.8 |
| Lung abscess or empyema | CD/DD | | 510, 513 |
| Cholecystitis | CD/DD | | 574.0-574.1,574.3-574.4, 574.6-574.8, 575.0-575.1 |
| Arteriovenous shunt infection | CD/DD | | 996.62, 996.63 |
| PD related peritonitis | CD/DD | | 567, 996.68, 999.3 |
| Shock | CD/DD | | 785.5, 785.51, 785.59 |
| Out-of-hospital cardiac arrest | CD | | 798, 798.1, 798.2, 798.9 |

Abbreviation: ICD-9-CM, International Classification of Disease, 9^th^ Revision, Clinical Modification

^#^Data source included CD (Ambulatory or emergency care expenditures by visits), HV (Registry for catastrophic illness patients), and DD (Inpatient expenditures by admissions). Detailed information can be accessed at <http://nhird.nhri.org.tw/date_04.html>

**Table B. The payment codes for the clinical treatment providing by Taiwan National Health Insurance**

| **Clinical treatment** | **Database^#^** | **Corresponding payment codes^$^** |
| --- | --- | --- |
| Hemodialysis treatment per patient visit | OO | 58001C, 58019C, 58020C, 58021C, 58022C, 58023C, 58024C, 58025C, 58027C, and 58029C. |
| Peritoneal dialysis solution | OO | B022198209, B022198210, B022198212, B022198214, B022198221, B022199209, B022199210, B022199212, B022199214, B022199221, B022218209, B022218210, B022218212, B022218214, B022218221, B022297210, B022297212, B022297214, B022297221, B022298214, B024219212, B023687212, B018239209, B018239210, B018239212, B018239214, B018239221, B018239277, B018239299, B018240209, B018240210, B018240212, B018240214, B018240221, B018240299, B018241209, B018241210, B018241212, B018241214, B018241221, B018241277, B020803210, B020803212, B020803214, B020803221, B020805210, B020805212, B020805214, B020805221, B020806210, B020806212, B020806214, B020806221, B022298210, B022298212, B022298221, B022299210, B022299212, B022299214, B022299221, B022915212, B023901214, B023901216, B023906216, B023907214, B023907216, B017312210, B017312212, B017312221, B017312299, B018196210, B018196212, B018196214, B018196221, B018196299, B022853212, B022853214, B022853221, B022854212, B022854214, B022854221, B022855212, B022855214, B022855221, B024829210, B024829212, B024829214, B024829221, B024830210, B024830212, B024830214, B024830221, B024831212, B024831214, B024831221, B024920212, B024921212, B024922212, B017867210, B017867212, B017867214, B017867221, B017867299, B023901212, B023906212, B023907212, B017312209, B017312277, B018196209, B018196277, B017867209, B017867277, B024377212, B024377214, B024378212, B024378214, B024414212, B024414214, B024418212, B024418214, B024419212, B024419214, B024420212, B024420214 |
| Arteriovenous fistula | OO | 69032C, 69032B, 69038C, 69032BB |
| Catheter | OO | CKDD2D3500BA, CKDD2D3500BA, CKDD2D3500VC, CKDD2D4000BA, CKDD2D4000BA, CKDD2D4000VC, CKDD2D4400BA, CKDD2D4400BA, CKDD2D4400VC, CKDD2D4424VC, CKDD2J0KADJ0, CKDD2J0KADJ0,  CKDD2K4400BA, CKDD2K4400BA, CKDD2K4400VC, CKDD2L4424VC, CKDD2L4424VC, CKDD2SL12PM0, CKDD2SL12PM0, CKDD2SL18PM0, CKDD2SL18PM0, CKDD312123AR, CKDD312123AR, CKDD323334BQ, CKDD323334BQ, CKDD325123AR, CKDD325123AR, CKDD3456032C, CKDD3456032C, CKDD345603KD, |
| Cardiopulmonary resuscitation | OO | 47029C |
| **Laboratory examination** | | |
| Creatinine (estimated glomerular filtration rate) | OO | 09015C |
| Potassium | OO | 09022C |
| Calcium | OO | 24007A, 24007B |
| Phosphorus | OO | 09012C |

^#^Data source included OO (Details of ambulatory care orders). Detailed information can be accessed at <http://nhird.nhri.org.tw/date_04.html>

^$^Information can be accessed at <http://www.nhi.gov.tw/query/query2.aspx?menu=20&menu_id=712&WD_ID=830> and <http://www.nhi.gov.tw/Query/query1.aspx?menu=20&menu_id=712&WD_ID=831>

**Table C. Anatomical Therapeutic Chemical codes of drugs used concomitantly by patients during the study period**

| **Drug type** | **Database^#^** | **ATC classification system codes** | **Drug name** |
| --- | --- | --- | --- |
| **Diabetic drugs** |  |  |  |
| Oral antidiabetic agents | OO | A10B | Acarbose, Acetohexamide, Buformin, Chlorpropamide, Gliclazide, Glimepiride, Glipizide, Gliquidone, Glyburide, Metformin, Nateglinide, Pioglitazone, Repaglinide, Rosiglitazone, Tolazamide, Tolbutamide, Sitagliptin, Miglitol |
| Insulin | OO | A10A | Insulin human, Insulin zinc crystal, Insulin chromatograp, Insulin monocomponem, Insulin isophane, Insulin protamine, Insulin lispro, Insulin glargine, Insulin aspart, Insulin glulisine, Insulin detemir |
| **Antihypertensive drugs** |  |  |  |
| Diuretics | OO | C03 | Thiazide (Hydrochlorothiazide, Trichlormethiazide), Furosemide, Amiloride, Spironolactone, Triamterene |
| Angiotensin-converting enzyme inhibitors (ACEIs) | OO | C09A, C09B | Captopril, Enalapril, Lisinopril, Perindopril, Ramipril, Quinapril, Benazepril, Cilazapril, Fosinopril, Imidapril |
| Angiotensin receptor blockers (ARBs) | OO | C09C, C09D | Candesartan, Irbesartan, Losartan, Olmesartan, Telmisartan, Valsartan |
| Alfa-blocker | OO | C02CA | Tamsulosin, Doxazosin, Terazosin |
| Beta-blockers | OO | C07A | Labetalol, Pindolol, Acebutolol, Alprenolol, Atenolol, Betaxolol, Bisoprolol, Carteolol, Carvedilol, Nadolol, Metoprolol, Oxprenolol, Propranolol, Sotalol, Timolol, Metipranolol, Esmolol |
| Calcium channel blockers | OO | C08C, C08D, C08E | Nifedipine, Nicardipine, Felodipine, Amlopidine, Isradipine, Diltiazem, Verapamil |
| Central-acting agent | OO | C02AC01, C02CC02, C02AB01, C02AB02 | Clonidine HCL, Guanethidine sulfate, Methyldopa |
| Vasodilator | OO | C04A | Hydralazine, Reserpine |
| **Analgesic drugs** |  |  |  |
| NSAIDs | OO | M01AA, M01AB, M01AC, M01AE, M01AG, M01AX | Aceclofenac, Acemetacin, Alclofenac, Benzydamine, Diclofenac, Etodolac, Fenbufen, Fenoprofen, Flufenamic acid, Flurbiprofen,Ibuprofen, Indomethacin, Ketoprofen, Ketorolac, Mefenamic acid, Meloxicam, Nabumetone, Naproxen, Niflumic acid, Nimesulide, Phenylbutazone, Piroxicam, Sulindac, Tenoxicam, Tiaprofenic acid, Tolfenamic acid, Tolmetin |
| **Anti-lipid drugs** |  |  |  |
| Statins | OO | C10AA | Atorvastatin, Fluvastatin, Lovastatin, Pravastatin, Rosuvastatin, Simvastatin |
| Fibrate | OO | C10AB | Bezafibrate, Clofibrate, Etofibrate, Gemfibrozil, Fenofibrate, Simfibrate |
| Other anti-lipid agents | OO | C10AD06, C10AC01, C10AC02, C10BA05, C10AD01, C10AD-, C10AX02 | Acipimox, Cholestyramine, Colestipol HCL, Ezetimibe, Niceritrol, Nicomol, Probucol |
| Erythropoietins | OO | B03XA01 | [Darbepoetin alfa](https://en.wikipedia.org/wiki/Darbepoetin_alfa), [Darbepoetin](https://en.wikipedia.org/wiki/Darbepoetin_alfa) beta, [Methoxy polyethylene glycol-epoetin beta](https://en.wikipedia.org/wiki/Methoxy_polyethylene_glycol-epoetin_beta) |

^#^Data source included OO (Details of ambulatory care orders). Detailed information can be accessed at <http://nhird.nhri.org.tw/date_04.html>

**Figure A. Study flow diagram**.


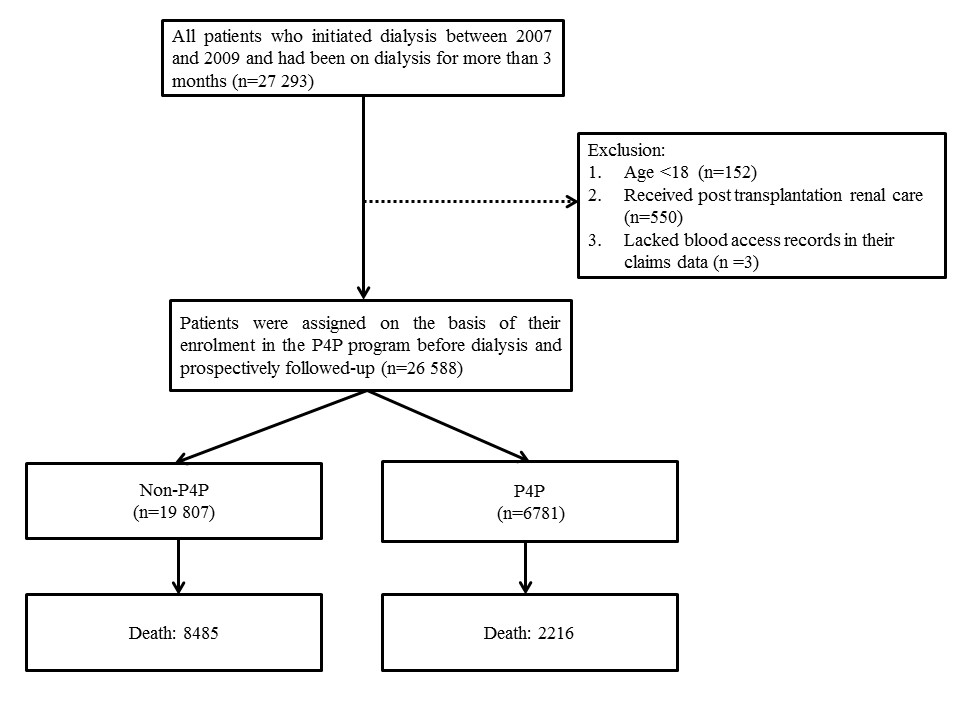

Supplement: S1 File — (DOCX) [file pone.0198387.s001.docx]
